# Classification of self-care patterns in Korean adults with prediabetes using unsupervised machine learning: a secondary data analysis

**DOI:** 10.7586/jkbns.25.060

**Published:** 2025-11-18

**Authors:** Mi-Kyoung Cho, Myoung-Lyun Heo

**Affiliations:** 1 Department of Nursing Science, College of Nursing, Chungbuk National University, Cheongju, Korea; 2 Department of Nursing, Jeonju University, Jeonju, Korea

**Keywords:** Nursing, Self care, Prediabetic state, Machine learning, Cluster analysis

## Abstract

**Purpose:**

This study aimed to classify self-care patterns among Korean adults with prediabetes using an unsupervised machine learning approach. The classification was grounded in Orem’s Self-Care Theory, focusing on self-care demands, self-care agencies, and self-care behaviors.

**Methods:**

A secondary data analysis was conducted using the 2023 Korea National Health and Nutrition Examination Survey. Variables were selected and categorized according to the theoretical components of Orem’s model. Principal component analysis was applied for dimensionality reduction, followed by K-means clustering to identify distinct self-care pattern groups. All variables were standardized using min–max normalization. Group differences were examined using analysis of variance and the chi-square test.

**Results:**

Three self-care pattern groups were identified: the high self-care performance group, the latent self-care risk group, and the self-care vulnerable group. These groups exhibited distinct profiles across self-care demands, agencies, and behaviors. Significant intergroup differences were also observed in education level, income, health literacy, fasting blood glucose, and hemoglobin A1c levels.

**Conclusion:**

Self-care patterns among adults with prediabetes can be effectively classified through unsupervised learning techniques. The findings highlight the importance of developing tailored nursing interventions that consider multidimensional self-care profiles. This study underscores the applicability of Orem’s Self-Care Theory and demonstrates the potential of machine learning in identifying at-risk subgroups for early intervention.

## INTRODUCTION

The global prevalence of diabetes is increasing due to changes in diet and lifestyle. In South Korea, 15.5% of adults aged ≥ 30 years had diabetes and 41.1% had prediabetes in 2021~2022, highlighting a critical window for early lifestyle-based interventions [1]. Prediabetes is clearly distinct from normal glycemic states and is characterized by increased insulin resistance and impaired glucose regulation, which already heighten the risk for both microvascular and macrovascular complications [2]. Without proactive management at the prediabetes stage, individuals may experience decreased quality of life and increased personal and societal healthcare expenditures [3,4].

For individuals with prediabetes, lifestyle modifications such as dietary control, physical activity, and weight reduction are considered the most effective preventive strategies, rather than pharmacological interventions [5]. The American Diabetes Association (ADA) recommends that adults with prediabetes engage in at least 150 minutes of moderate-intensity aerobic exercise per week and achieve weight loss, as such behavioral improvements enhance insulin sensitivity and directly contribute to better glycemic control [6]. Meanwhile, the practice of self-care behaviors is closely linked to individual capacities, such as educational attainment and health literacy [7]. In fact, individuals with higher health literacy tend to acquire necessary information for diabetes management more effectively and translate it into consistent self-care behaviors [8]. Therefore, nursing strategies aimed at diabetes prevention should extend beyond general education and instead focus on identifying levels and patterns of self-care among individuals, enabling the development of tailored interventions suited to specific needs.

While the significance of self-care in diabetes prevention has been well established in prior studies, a clear theoretical framework is crucial for a systematic understanding of self-care behaviors. Orem's Self-Care Deficit Nursing Theory offers a comprehensive explanation of health management, focusing on the interaction between self-care demands, self-care agency, and self-care behaviors. The theory defines a self-care deficit as a mismatch between an individual's ability to perform self-care and the necessary actions required to maintain health [9]. Research using this theory in chronic disease management, including diabetes and hypertension, has shown that individuals with higher self-care agency are more likely to adopt health-promoting behaviors, such as maintaining a healthy diet and engaging in regular physical activity, which ultimately lead to improved health outcomes [10,11].

Although prediabetes is a stage preceding the onset of disease, it is marked by increasing insulin resistance and a heightened need for self-care [2]. During this critical period, self-care behaviors among adults with prediabetes can be conceptualized not merely as the presence or absence of action, but as typologies emerging from the complex interplay of various factors, as explained by Orem's Self-Care Theory. Classifying these behavioral patterns provides insight into why certain subgroups, despite being at high risk, fail to adequately engage in self-care—reflecting a mismatch between self-care demands and behaviors. Moreover, such typological classification facilitates the design of tailored nursing interventions that are aligned with the specific characteristics of each subgroup [12]. Thus, Orem's theory serves as an appropriate and robust conceptual framework for analyzing and typifying the multifaceted self-care behaviors observed in adults with prediabetes.

Recently, interest in prediabetes prevention and management has gained momentum, as evidenced by a bibliometric analysis highlighting escalating global research trends in this area [13]. However, existing studies predominantly rely on traditional statistical methods, providing fragmented analyses of influencing factors [14]. Such approaches often fail to uncover latent heterogeneity in complex health behavior data or to elucidate individualized self‑care typologies. Moreover, there is a notable lack of theory-based investigations that could contribute to the systematic conceptualization of self‑care behaviors. Consequently, a conceptually grounded framework is needed to advance our understanding and classification of self‑care patterns in prediabetes.

This study aims to scientifically classify self-care typologies among Korean adults with prediabetes by applying an unsupervised machine learning technique that captures nonlinear interactions within complex data, using the large-scale, nationally representative Korea National Health and Nutrition Examination Survey (KNHANES) dataset [15]. By integrating Orem's Self-Care Theory into the exploration of typologies, this research enhances its theoretical validity and seeks to provide evidence for personalized nursing interventions applicable in clinical practice. Therefore, the objective of this study is to identify and interpret self-care patterns among adults with prediabetes using exploratory clustering techniques based on machine learning, and to contextualize the results within the framework of Orem's theory. The findings are expected to serve as foundational data for developing tailored diabetes prevention strategies according to distinct self-care typologies.

## METHODS

### 1. Study design

This study is a secondary data analysis that employs machine learning to classify self-care types among adults with prediabetes, using raw data from the 2nd year of the 9th Korea KNHANES, conducted in 2023.

### 2. Participants

This study targeted adults aged 19 to 64 years who were identified as having prediabetes based on the raw data from the second year (2023) of the 9th KNHANES [16]. Prediabetes was defined according to the classification criteria provided in the KNHANES guidelines, which are based on the diagnostic standards of ADA [6]. Specifically, individuals with a fasting blood sugar level of 100~125 mg/dL or a glycated hemoglobin (HbA1c) level of 5.7%~6.4% were classified as having prediabetes [6,16]. Of the total 6,929 survey respondents, 4,071 were adults aged 19~64 years. Among them, 1,051 individuals met either of the two criteria for prediabetes and were initially selected for analysis. Since machine learning analysis requires complete data across all input variables, individuals with any missing values in the study variables were excluded from the analysis. Consequently, a final sample of 931 participants with complete data was included in the analysis.

### 3. Instruments

#### 1) Self-care components

In this study, the components of self-care were categorized into self-care demands, self-care agencies, and self-care behaviors based on Orem's Self-Care Theory. Variables corresponding to each component were selected according to their theoretical definitions and supported by evidence from previous studies.

##### (1) Self-care demands

Self-care demands refer to the conditions that individuals must meet to maintain health or prevent and manage disease, as defined in nursing theory. In the context of diabetes management, fasting blood sugar (FBS, mg/dL) and HbA1c (%) are key biochemical indicators used to assess the need for diagnosis and intervention in both diabetes and prediabetes [17]. These indicators reflect the necessity for self-care interventions. Accordingly, this study included FBS and HbA1c levels as continuous variables to represent self-care demands.

##### (2) Self-care agencies

Self-care agencies refer to an individual's ability to recognize their self-care needs and to take the necessary actions to meet those needs, as defined in nursing theory. In the context of diabetes management, educational attainment, financial resources, and health literacy are foundational elements that enhance self-management capacity. Prior studies have reported that these factors are closely associated with blood glucose control and successful self-management outcomes [18]. To capture self-care agency in this study, the following variables were included: education level (0 = high school graduate or less, 1 = some college or higher) and individual income (0 = upper-middle level and above, 1 = lower-middle level and below). Health literacy, defined as the cognitive ability to seek, understand, evaluate, and apply health-related information to actual health behaviors, was assessed using a validated instrument developed by Yoon et al. [19] for the KNHANES. This tool consists of 10 items rated on a 4-point Likert scale, covering four domains: disease prevention, health promotion, health management, and resource utilization. The reliability of the instrument was assessed using Cronbach's α, which was .87 at the time of development [19] and .99 in the current study.

##### (3) Self-care behaviors

Self-care behaviors refer to concrete actions performed in daily life based on an individual's self-care agency, as conceptualized in nursing theory. In diabetes management, self-care behaviors are crucial for achieving glycemic control and improving metabolic health, and their significance has been consistently emphasized in clinical and epidemiological research [6]. To assess self-care behaviors in this study, the following lifestyle-related indicators were included: engagement in aerobic activity (0 = no, 1 = yes), engagement in muscle-strengthening exercise (0 = no, 1 = at least one day), average weekday sleep (hours), and dietary control (0 = no, 1 = yes). These variables represent key behavioral practices commonly associated with the effective prevention and management of diabetes.

#### 2) Participant characteristics

Participant characteristics were analyzed based on the fundamental conditioning factors outlined in Orem's Self-Care Theory. Age was treated as a continuous variable, while categorical sociodemographic variables—such as sex, marital status, and single-person household status—were coded as dummy variables (as per nursing research conventions).

Physical characteristics included waist circumference (cm) and body mass index (BMI), with BMI dichotomized as < 23 kg/m² = 0 and ≥ 23 kg/m² = 1, a widely used threshold for identifying obesity and metabolic disease risk [20]. Additionally, two clinical conditions known to be strongly associated with diabetes-related health outcomes were included as control variables: hypertension (coded as 0 = normal, 1 = elevated blood pressure, 2 = hypertension) and hypercholesterolemia (0 = no, 1 = yes), based on prior evidence in diabetes care research [21].

### 4. Statistical analysis

Data analysis was conducted using SPSS version 28.0 (IBM Corp., Armonk, NY, USA) and Orange version 3.4 (University of Ljubljana, Ljubljana, Slovenia), a machine learning toolkit. The analysis comprised two main phases: unsupervised machine learning and comparison of cluster characteristics.

#### 1) Unsupervised learning workflow

**Figure 1.**
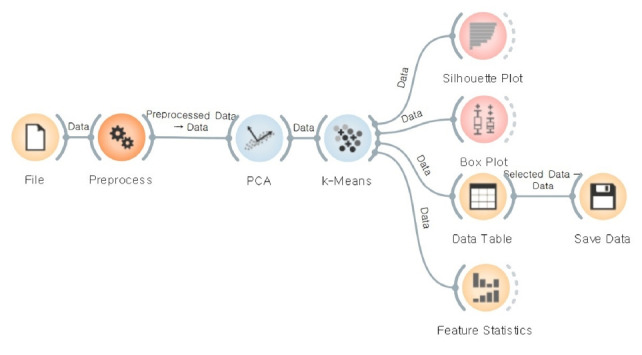
Workflow of clustering analysis using Orange. PCA = Principal component analysis.

Data preprocessing was conducted using SPSS version 28.0 and Orange version 3.4. First, cases with missing values were removed. All variables were then transformed into numerical vectors to enable processing by machine learning algorithms. Nominal variables were converted into dummy variables through one-hot encoding, while ordinal variables were encoded using ordinal encoding to preserve rank information. All variables were treated as continuous and standardized using Z-score normalization to adjust for differences in scale, which is essential for distance-based analyses. Subsequently, principal component analysis (PCA) was performed using the PCA widget in Orange. Default settings were applied, and six principal components were extracted to achieve a cumulative explained variance of over 70%, in accordance with established guidelines [22]. These six components accounted for approximately 74% of the total variance in the dataset. The dimension-reduced data were then input into the K-means widget for clustering. Although various methods exist for determining the optimal number of clusters (K), the analyst must ultimately specify a value for K. In this study, K was manually set to 3 based on prior research by Zupa (2021) [23], which categorized patients into high-, moderate-, and low-risk groups for glycemic control. The initial seed value was kept at the default, iteration settings were automatic, and Euclidean distance was used as the distance metric. Clustering validity was assessed using the Silhouette plot widget. The silhouette score is a numerical measure of the separation between clusters and the cohesion within clusters [24]. It was used in this study to evaluate the appropriateness of the clustering structure. Generally, silhouette scores close to 1 indicate well-defined clusters with strong internal cohesion, while scores above 0.5 suggest a meaningful clustering structure [25]. The workflow for this analysis is illustrated in Figure 1.

#### 2) Comparison of cluster characteristics

Although this study utilized data from the KNHANES, machine learning approaches based on unsupervised learning are not suitable for incorporating the complex sampling design (i.e., stratification, clustering, and sample weights). Therefore, cluster classification and interpretation were performed using a simplified analytic approach that did not account for the complex survey design. To compare characteristics across clusters, SPSS was used to perform frequency analysis and descriptive statistics. Cross-tabulations were conducted to examine differences in the distribution of categorical variables among clusters. For continuous variables, one-way analysis of variance was used to assess mean differences across clusters, followed by Scheffé's post hoc test for pairwise comparisons.

### 5. Ethical considerations

This study utilized anonymized secondary data collected for public health purposes, and the researchers had no access to personally identifiable information. The data were drawn from the raw dataset of the 2nd year of the 9th KNHANES, conducted in 2023 by the Korea Disease Control and Prevention Agency (KDCA). The KNHANES received ethical approval from the Institutional Review Board under the KDCA, and all participants provided written informed consent at the time of data collection.

## RESULTS

### 1. General and self-care characteristics of participants

Analysis of the participants' general characteristics revealed a mean age of 50.76 ± 10.42 years. Of the participants, 450 (48.3%) were male, 803 (86.3%) were married, and 810 (87.0%) lived in households with two or more members. Regarding physical health indicators, 660 participants (70.9%) had a BMI of 23 kg/m² or greater, 319 (34.3%) had hypercholesterolemia, 270 (29.0%) were classified as having elevated blood pressure, and 289 (31.0%) had hypertension. The mean waist circumference was 87.11 ± 10.29 cm. In terms of self-care demands, the mean FBS was 103.03 ± 7.79 mg/dL, and the mean HbA1c was 5.61 ± 0.31%. For self-care agencies, 52.2% of participants had a high school education or less, while individual income was nearly evenly divided between upper-middle level and above (50.2%) and lower-middle level and below (49.8%). The mean health literacy score was 30.53 ± 4.90. Regarding self-care behaviors, 48.7% engaged in aerobic exercise, 28.8% performed muscle-strengthening exercise at least once a week, and 29.6% practiced dietary control. The average weekday sleep duration was 6.59 ± 1.20 hours (Table 1).

### 2. Cluster identification using unsupervised PCA–K-means analysis

**Figure 2.**
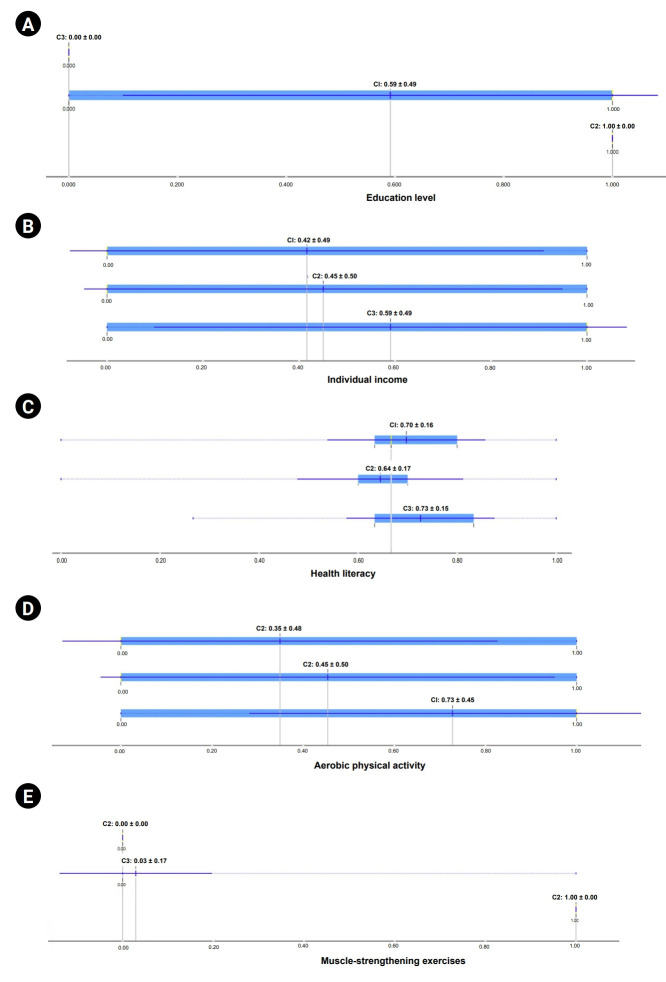
Inter-cluster differences in self-care-related variables. (A) education level, (B) individual income, (C) health literacy, (D) aerobic physical activity, and (E) muscle-strengthening activity. C = Cluster.

Unsupervised PCA–K-means clustering was conducted using Orange, a machine learning–based data mining tool. Six principal components explaining 74.0% of the total variance were extracted through PCA, and these components were subsequently used as input for the K-means algorithm. As a result, three distinct clusters were identified (Table 2). The degree of separation between clusters was evaluated using silhouette scores. Based on normalized data, the silhouette scores for all three clusters exceeded 0.5, indicating an overall adequate level of separation (F = 22.25, *p* < .001). The mean and standard deviation for each variable within the clusters were calculated using the normalized data of participants in each group, reflecting relative distributional differences across variables. The analysis revealed statistically significant differences among clusters for all variables except average weekday sleep duration. In particular, the following variables showed the most pronounced inter-cluster differences: education level (F = 1271.66, *p* < .001, Figure 2-A), individual income (F = 11.52, *p* < .001, Figure 2-B), health literacy (F = 23.30, *p* < .001, Figure 2-C), engagement in aerobic physical activity (F = 49.57, *p* < .001, Figure 2-D), and muscle-strengthening activity (F = 7825.86, *p* < .001, Figure 2-E). These variables were visualized using box plots to illustrate distributional patterns across clusters.

### 3. Group differences in moderating factors and self-care characteristics

To identify differences in moderating factors and self-care characteristics across the three clusters (C1, C2, C3) derived via unsupervised learning-based cluster analysis, statistical group comparisons were conducted (Table 3). Among the general moderating factors, age was highest in Cluster 3 (F = 27.64, *p* < .001), and the proportion of men was highest in Cluster 1 (χ² = 30.57, *p* < .001). Marital status also differed significantly across clusters, with the highest proportion of married individuals in Cluster 3 (χ² = 13.19, *p* = .001). Similarly, the prevalence of elevated blood pressure and hypertension was most significant in Cluster 3 (χ² = 34.09, *p* < .001). In contrast, there were no significant differences across clusters in single-person households, hypercholesterolemia status, BMI, or waist circumference. Regarding self-care needs, both fasting blood glucose (F = 3.44, *p* = .033) and HbA1c (F = 5.54, *p* = .004) were highest in Cluster 3. Post hoc analysis indicated that Cluster 3 had significantly higher levels than Clusters 1 and 2. In terms of self-care capacity, significant differences were observed across clusters for education level (χ² = 682.11, *p* < .001) and personal income (χ² = 22.55, *p* < .001). Education levels were higher in Clusters 1 and 2 than in Cluster 3, while individual income was highest in Cluster 1. Health literacy also showed significant group differences (F = 23.30, *p* < .001), with post hoc tests revealing a decreasing trend from Cluster 1 to Cluster 3. In terms of self-care behaviors, except for weekday sleep duration, significant differences were found across clusters for engagement in aerobic exercise (χ² = 89.86, *p* < .001), muscle-strengthening exercise (χ² = 878.89, *p* < .001), and dietary control (χ² = 12.42, *p* = .002), with Cluster 1 consistently demonstrating the highest proportions of adherence.

### 4. Cluster labeling based on self-care characteristics

In this study, the three clusters derived from unsupervised clustering analysis were labeled based on the core components of Orem's Self-Care Theory—self-care needs, self-care agency, and self-care behaviors. Low self-care demands, high levels of education and individual income, and strong health literacy characterized cluster 1 (C1). Participants in this cluster also showed high levels of engagement in self-care behaviors, including aerobic exercise, muscle-strengthening exercise, and dietary control. Based on these characteristics, this group was labeled the "High Self-Care Performance Group." Cluster 2 (C2) also exhibited low self-care demands and a high level of education, but had lower individual income and moderate health literacy. Despite a sufficient educational background, participants in this group showed low engagement in aerobic exercise and dietary control, with particularly low levels of muscle-strengthening exercise. Given this mixed profile, this cluster was labeled the "Latent Risk Group for Self-Care." Cluster 3 (C3) had the highest level of self-care demands, yet exhibited the lowest levels of education, individual income, and health literacy. In addition, participants in this cluster showed the poorest adherence to self-care behaviors across all measured domains. Accordingly, this cluster was designated the "Vulnerable Group for Self-Care".

## DISCUSSION

Using data from the 9th cycle, 2nd year of the KNHANES (2023), this study conducted unsupervised machine learning–based clustering analysis on prediabetic adults, focusing on Orem's self-care components—self-care demand, self-care agency, and self-care behaviors. Based on the relative levels of these attributes, three distinct self-care profiles were identified and labeled as the High Self-Care Performance Group, the Latent Self-Care Risk Group, and the Self-Care Vulnerable Group. The following discussion outlines tailored nursing intervention strategies for each group.

The High Self-Care Performance Group showed low levels of self-care demand, yet high levels of education, income, and health literacy. This group consistently engaged in recommended self-care behaviors, including aerobic exercise, muscle-strengthening exercise, and dietary control. Interventions for this group should focus on maintaining current behavior while preventing burnout or motivational fatigue. Garner et al. [26] found that volunteers who participated in a diabetes lifestyle mentoring program experienced improved dietary habits, reduced sedentary time, and significantly enhanced diabetes-specific self-efficacy. Therefore, members of this group may also benefit from serving as peer mentors, which can reinforce self-care among mentees while simultaneously helping to prevent burnout in mentors.

The Latent Self-Care Risk Group was characterized by low self-care demand, relatively high educational attainment, but lower income, and moderate health literacy. Despite having adequate informational capacity, individuals in this group exhibited low adherence to self-care behaviors, particularly in muscle-strengthening exercise. These findings suggest that merely providing knowledge is insufficient; interventions should also include practical training in behavioral skills. Rahayu [27] highlighted the mediating role of self-efficacy between diabetes knowledge and self-care behavior, underscoring the need to enhance self-efficacy. Effective strategies may include goal-setting, practice and tracking of self-management skills, peer modeling, and positive reinforcement [28].

The Self-Care Vulnerable Group had the highest self-care demand, but also the lowest levels of education, income, health literacy, and behavioral adherence. These individuals may struggle with self-care due to limited information processing capacity and constrained social resources. Educational interventions tailored to their level of health literacy, hands-on training to facilitate behavior change, and support systems involving family and community networks are essential [29,30]. Given that this group is also affected by social determinants of health, such as low income and limited access to care [31], structural support through community-based and national policy-level interventions is crucial.

Taken together, the findings demonstrate that self-care levels among prediabetic adults are shaped not by a single variable, but by the interaction of multiple factors. Notably, general demographic variables such as sex, age, and marital status did not significantly differ across groups, indicating that self-care is more directly influenced by health literacy, educational attainment, behavioral capability, and self-care demand. The limited self-care capacity observed in the vulnerable group may be due to restricted access to information and education, which reinforces Orem's assertion that nursing plays a critical role in supporting individuals in meeting their self-care demands [32]. This study contributes to theoretical development by demonstrating the need for group-specific interventions at the prediabetic stage.

Methodologically, this study employed unsupervised clustering, a machine learning technique that identifies hidden patterns in data without pre-labeled outcomes. This allowed for the discovery of latent self-care types that may be overlooked in traditional variable-driven analyses, enabling early identification of at-risk individuals in clinical practice [33]. Because the core variables used in the clustering process can be collected via simple questionnaires, the resulting model has the potential to be operationalized as a quick screening tool in clinical and community settings. From an academic perspective, this study contributes to the development of refined, tailored preventive nursing interventions by classifying individuals based on integrated dimensions of self-care demand, agency, and behavior.

Nevertheless, the study has some limitations. The analysis did not incorporate the complex sampling design of the KNHANES dataset, and, as an exploratory clustering study, it did not utilize advanced machine learning features, such as hyperparameter tuning. Still, the Orange software used is known for its accuracy and user-friendly interface, allowing non-programmers to perform intuitive clustering analysis suitable for exploratory research [34]. Future research should aim to replicate and validate these findings, develop predictive tools for cluster classification, evaluate the effects of interventions across identified groups, and compare alternative machine learning algorithms to enhance generalizability and practical application.

## CONCLUSION

Grounded in Orem's Self-Care Theory, this study examined the self-care characteristics of adults with prediabetes, focusing on three core components: self-care demand, self-care agency, and self-care behavior. Using an unsupervised learning approach—specifically PCA-K-means clustering—participants were categorized into three distinct groups: the High Self-Care Performance Group, the Latent Self-Care Risk Group, and the Self-Care Vulnerable Group. The findings revealed that differences in self-care agency and behavior were more pronounced than those based on sociodemographic characteristics. These results support Orem's theoretical assertion that nursing plays a facilitative role in enhancing individuals' capacity to meet their own self-care demands. Moreover, the findings demonstrate that self-care capacity is not determined by a single factor, but rather by the complex interaction of multiple dimensions, underscoring the need for tailored nursing interventions. Importantly, by applying an unsupervised machine learning technique, the study was able to uncover latent subgroups that may have been overlooked in prior research, highlighting the potential for early intervention in both clinical and community health settings. Based on these findings, it is recommended that adults with prediabetes be clustered using simple biological indicators such as blood glucose and HbA1c in combination with basic self-care variables, to facilitate the development and application of tailored nursing interventions for each subgroup. Nonetheless, this study has limitations. The complex sampling design of the KNHANES was not fully accounted for, which may limit the generalizability of the findings. In addition, the clustering analysis remained exploratory, without extending to model validation or optimization. Future research should aim to replicate these findings using diverse datasets, develop predictive indicators for self-care typologies, evaluate the effectiveness of targeted nursing interventions, and explore the comparative utility of various machine learning techniques to enhance both practical applicability and generalizability.

## Figures and Tables

**Table 1.** General and Self-care Characteristics of Participants (N = 931)

Characteristics			n (%) or M ± SD
General regulatory factors	Age (years)		50.76 ± 10.42
	Sex	Men	450 (48.3)
		Women	481 (51.7)
	Marital status	Married	803 (86.3)
		Single	128 (13.7)
	Single-person household	Yes	121 (13.0)
		No	810 (87.0)
	BMI (kg/m²)	< 23	271 (29.1)
		≥ 23	660 (70.9)
	Hypercholesterolemia	Yes	319 (34.3)
		No	612 (65.7)
	Hypertension	Normal	372 (40.0)
		Elevated blood pressure	270 (29.0)
		Hypertension	289 (31.0)
	Waist circumference		87.11 ± 10.29
Self-care demands	FBS (mg/dL)		103.03 ± 7.79
	HbA1c (%)		5.61 ± 0.31
Self-care agencies	Education level	High school graduate or less	486 (52.2)
		Some college or higher	445 (47.8)
	Individual income	Upper-middle level and above	467 (50.2)
		Lower-middle level and below	464 (49.8)
	Health literacy		30.53 ± 4.90
Self-care behaviors	Aerobic exercise	No	478 (51.3)
		Yes	453 (48.7)
	Muscle-strengthening exercise	No	663 (71.2)
		At least one day	268 (28.8)
	Diet control	No	655 (70.4)
		Yes	276 (29.6)
	Weekday sleep (hours)		6.59 ± 1.20

M = Mean; SD = Standard deviation; BMI = Body mass index; FBS = Fasting blood sugar; HbA1c = Glycated hemoglobin.

**Table 2.** Cluster Identification Using Unsupervised PCA–K-means Analysis

Types of self-care/Variables		C1	C2	C3	ANOVA	
		M ± SD	M ± SD	M ± SD	F	*p*
Self-care demands	FBS (mg/dL)	0.56 ± 0.15	0.53 ± 0.16	0.56 ± 0.16	3.44	.033
	HbA1c (%)	0.55 ± 0.17	0.55 ± 0.17	0.59 ± 0.17	5.54	.004
Self-care agencies	Education level	0.59 ± 0.49	1.00 ± 0.00	0.00 ± 0.00	1271.66	< .001
	Individual income	0.42 ± 0.49	0.45 ± 0.50	0.59 ± 0.49	11.52	< .001
	Health literacy	0.70 ± 0.16	0.64 ± 0.17	0.73 ± 0.15	23.30	< .001
Self-care behaviors	Aerobic exercise	0.73 ± 0.45	0.45 ± 0.50	0.35 ± 0.48	49.57	< .001
	Muscle-strengthening exercise	1.00 ± 0.00	0.00 ± 0.00	0.03 ± 0.17	7825.86	< .001
	Diet control	0.38 ± 0.49	0.26 ± 0.44	0.27 ± 0.44	6.28	.002
	Weekday sleep (hours)	0.40 ± 0.12	0.40 ± 0.13	0.40 ± 0.14	0.95	.054
Silhouette		0.54 ± 0.02	0.55 ± 0.01	0.54 ± 0.02	22.25	< .001
PCA: Components = 6, Explained variance = 74.0% (criteria > 60%)						
Silhouette (M ± SD) = .54 ± .14 (criteria > .50)						

All continuous variables were normalized to the 0~1 interval using min–max normalization, while binary variables were retained as 0/1 values. All values are presented as means and standard deviations for cluster analysis. PCA = Principal component analysis; C = Cluster; ANOVA = Analysis of variance; M = Mean; SD = Standard deviation; FBS = Fasting blood sugar; HbA1c = Glycated hemoglobin.

**Table 3.** Group Differences in Moderating Factors and Self-care Characteristics

Characteristics			C1 (n = 257)	C2 (n = 293)	C3 (n = 381)	χ² or F (*p*) (Scheffé test)
General regulatory factors	Age (years)		50.25 ± 11.18	47.67 ± 9.52	53.48 ± 9.85	27.64 (< .001) (C2 < C1 < C3)
	Sex	Men	151 (58.8)	155 (52.9)	144 (37.8)	30.57 (< .001)
		Women	106 (41.2)	138 (47.1)	237 (62.2)	
	Marital status	Married	216 (84.0)	240 (81.9)	347 (91.1)	13.19 (.001)
		Single	41 (16.0)	53 (18.1)	34 (8.9)	
	Single-person household	Yes	34 (13.2)	33 (11.3)	54 (14.2)	1.26 (.533)
		No	223 (86.8)	260 (88.7)	327 (85.8)	
	BMI (kg/m²)	< 23	80 (31.1)	86 (29.4)	105 (27.6)	0.96 (.619)
		≥ 23	177 (68.9)	207 (70.6)	276 (72.4)	
	Hypercholesterolemia	Yes	85 (33.1)	97 (33.1)	137 (36.0)	0.82 (.663)
		No	172 (66.9)	196 (66.9)	244 (64.0)	
	Hypertension	Normal	121 (47.1)	138 (47.1)	113 (29.7)	34.09 (< .001)
		Elevated blood pressure	67 (26.1)	86 (29.4)	117 (30.7)	
		Hypertension	69 (26.8)	69 (23.5)	151 (39.6)	
	Waist circumference (cm)		86.07 ± 10.52	87.13 ± 10.13	87.7 ± 10.21	2.16 (.116)
Self-care demands	FBS (mg/dL)		103.38 ± 7.58	102.05 ± 7.82	103.54 ± 7.85	3.44 (.033) (C1,C2 < C3)
	HbA1c (%)		5.59 ± 0.31	5.58 ± 0.31	5.65 ± 0.30	5.54 (.004) (C1,C2 < C3)
Self-care agencies	Education level	High school graduate or less	105 (40.9)	0 (0.0)	381 (100.0)	682.11 (< .001)
		Some college or higher	152 (59.1)	293 (100.0)	0 (0.0)	
	Individual income	Upper-middle level and above	150 (58.4)	161 (54.9)	156 (40.9)	22.55 (< .001)
		Lower-middle level and below	107 (41.6)	132 (45.1)	225 (59.1)	
	Health literacy		31.86 ± 4.45	30.92 ± 4.77	29.31 ± 5.00	23.30 (< .001) (C1 > C2 > C3)
Self-care behaviors	Aerobic exercise	No	70 (27.2)	160 (54.6)	248 (65.1)	89.86 (< .001)
		Yes	187 (72.8)	133 (45.4)	133 (34.9)	
	Muscle-strengthening exercise	No	0 (0.0)	293 (100.0)	370 (97.1)	878.89 (< .001)
		At least one day	257 (100.0)	0 (0.0)	11 (2.9)	
	Diet control	No	159 (61.9)	218 (74.4)	278 (73.0)	12.42 (.002)
		Yes	98 (38.1)	75 (25.6)	103 (27.0)	
	Weekday sleep (hours)		6.61 ± 1.07	6.57 ± 1.19	6.59 ± 1.29	0.05 (.948)

Values are presented as the mean ± standard deviation or n (%). C = Cluster; BMI = Body mass index; FBS = Fasting blood sugar; HbA1c = Glycated hemoglobin.

## Data Availability

The data that support the findings of this study are available from the corresponding author upon reasonable request.
